# Methyl 4-[*N*-(5-bromo­pyrimidin-2-yl)carbamo­yl]benzoate

**DOI:** 10.1107/S1600536812032102

**Published:** 2012-07-18

**Authors:** Hui-Ling Hu, Chia-Jun Wu, Chun-Wei Yeh, Jhy-Der Chen

**Affiliations:** a Department of Chemistry, Chung-Yuan Christian University, Chung-Li 320, Taiwan

## Abstract

In the title compound, C_13_H_10_BrN_3_O_3_, the pyrimidine and benzene rings are twisted with an inter­planar angle of 58.4 (1)°. The secondary amide group adopts a *cis* conformation with an H—N—C—O torsion angle of 14.8 (1)°. In the crystal, mol­ecules are connected into inversion dimers *via* pairs of N—H⋯N hydrogen bonds, generating an *R*_2_^2^(8) motif. The dimers are further connected through a C—Br⋯O inter­action [3.136 (1) Å and 169.31 (1)°] into a chain along [110]. Weak C—H⋯N hydrogen bonds between the methyl benzoate groups and pyrimidine rings are also observed in the crystal structure.

## Related literature

For methyl-4-(5-bromo­pyrimidin-2-ylcarbamo­yl)benzoate and its metal complexes, see: Wu *et al.* (2011 ▶). For the conformation of related amides, see Forbes *et al.* (2001 ▶); Oertli *et al.* (1992 ▶); Lu *et al.* (2011**a* ▶,b* ▶). For C—Br⋯O inter­actions, see: Rowland & Taylor (1996 ▶).
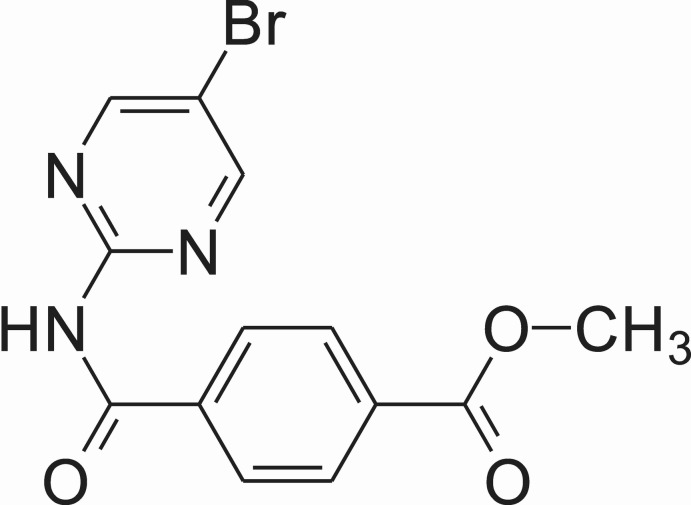


## Experimental

### 

#### Crystal data

C_13_H_10_BrN_3_O_3_
*M* *_r_* = 336.15
Triclinic, 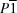

*a* = 5.9398 (6) Å
*b* = 7.4137 (7) Å
*c* = 15.897 (2) Å
α = 77.846 (9)°
β = 81.613 (7)°
γ = 68.185 (9)°
*V* = 633.58 (12) Å^3^
*Z* = 2
Mo *K*α radiation
μ = 3.26 mm^−1^
*T* = 295 K
0.4 × 0.3 × 0.2 mm

#### Data collection

Siemens P4 diffractometer
Absorption correction: ψ scan (*XSCANS*; Siemens, 1995 ▶) *T*_min_ = 0.953, *T*_max_ = 0.984
2880 measured reflections
2192 independent reflections
1841 reflections with *I* > 2σ(*I*)
*R*_int_ = 0.027
3 standard reflections every 97 reflections intensity decay: none

#### Refinement

*R*[*F* ^2^ > 2σ(*F* ^2^)] = 0.034
*wR*(*F* ^2^) = 0.076
*S* = 1.05
2192 reflections
186 parameters
H atoms treated by a mixture of independent and constrained refinement
Δρ_max_ = 0.30 e Å^−3^
Δρ_min_ = −0.41 e Å^−3^

### 

Data collection: *XSCANS* (Siemens, 1995 ▶); cell refinement: *XSCANS*; data reduction: *SHELXTL* (Sheldrick, 2008 ▶); program(s) used to solve structure: *SHELXS97* (Sheldrick, 2008 ▶); program(s) used to refine structure: *SHELXL97* (Sheldrick, 2008 ▶); molecular graphics: *SHELXTL*; software used to prepare material for publication: *SHELXTL*.

## Supplementary Material

Crystal structure: contains datablock(s) I, global. DOI: 10.1107/S1600536812032102/gw2122sup1.cif

Structure factors: contains datablock(s) I. DOI: 10.1107/S1600536812032102/gw2122Isup2.hkl

Supplementary material file. DOI: 10.1107/S1600536812032102/gw2122Isup3.cml

Additional supplementary materials:  crystallographic information; 3D view; checkCIF report

## Figures and Tables

**Table 1:** Hydrogen-bond geometry (Å, °)

*D*—H⋯*A*	*D*—H	H⋯*A*	*D*⋯*A*	*D*—H⋯*A*
N3—H3*A*⋯N1^i^	0.84 (4)	2.14 (1)	2.98 (1)	175 (1)
C13—H13*B*⋯N2^ii^	0.96	2.58	3.37 (1)	139

Symmetry codes: (i) 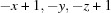; (ii) 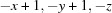.

**Fig. 1.**
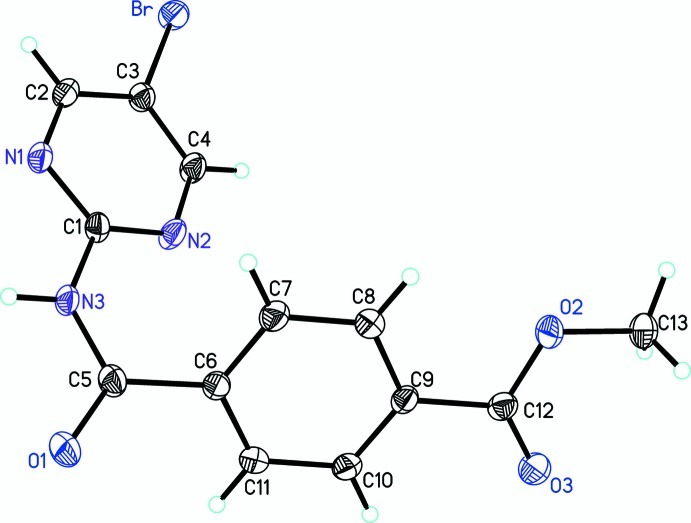
Crystal structure of the title compound with atom labeling and displacement ellipsoids drawn at the 30% probability level.

**Fig. 2.**
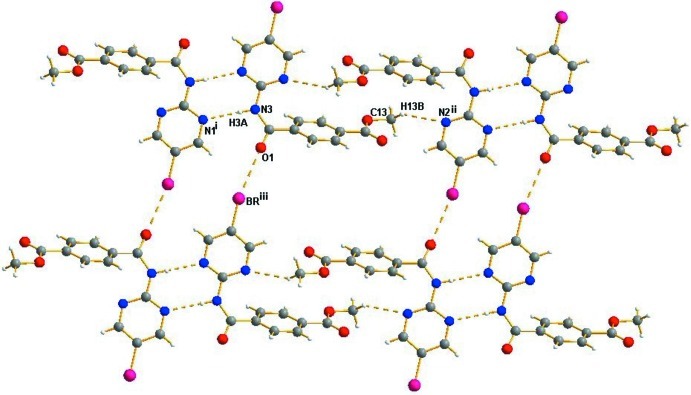
Partial packing diagram showing C—H···N and N—H···N hydrogen bonds and C—Br···O interactions among the molecule, with atom labeling. Symmetric code: (i) 1 - x, -y, 1 - z; (ii) 1 - x, 1 - y, -z; (iii) -1 + x, -1 + y, z.

## supplementary crystallographic information

### Comment

Several silver(I) complexes containg Methyl-4-(5-halopyrimidin-2-ylcarbamoyl)benzoate ligands have been reported, which show two-dimensional structures (Wu, *et al.*, 2011). Within this project the crystal structure of the title compound was determined (Fig.1). The pyrimidyl and phenyl rings are not coplanar but twisted with an interplanar angle of 58.4 (1)°. Several C—O lengths are found in the title compound for amide [C5—O1 = 1.220 (4) Å] and methyl benzoate groups [C12—O3 = 1.200 (4), C12—O2 = 1.335 (4) and C13—O2 = 1.448 (4) Å], and the C—N—C angles in pyrimidyl group [C1—N1—C2 = 116.1 (3) and C1—N2—C4 = 116.5 (3)°] is smaller than that in amide group [C1—N3—C5 = 131.2 (3)°]. In its crystal structure intermolecular N—H···N hydrogen bonds are found (Tab. 1) and the molecules are also interlinked through C—Br···O van der Waals interactions [3.136 (1) Å and 169.31 (1) °] (Rowland *et al.*, 1996). The weak C—H···N hydrogen bonds among the methyl benzoate and pyrimidyl rings are also found in the solid state (Fig. 2). In the crystal structure of the title compound the amide group adopts *cis* conformation with the H3A—N3—C5—O1 torsion angle of 14.8 (1) °, which is same as the chloro one (Lu, *et al.*, 2011*a*). This conformation is different from that in the Ag complex, which is *trans* (Wu, *et al.*, 2011; Lu, *et al.*, 2011*b*).

### Experimental

The title compound was prepared according to a published procedure (Wu *et al.*, 2011). Block crystals suitable for X-ray crystallography were obtained by slow evaporization of the solvent from a solution of the title compound in methanol.

### Refinement

H atoms bound to C atoms were placed in idealized positions and constrained to ride on their parent atoms, with C—H = 0.93 - 0.96 Å, and with *U*_iso_(H) = 1.2 or 1.5 *U*_eq_(C). The amine hydrogen atom (H3A) that is involved in the N—H···N hydrogen bond was freely refined.

### Crystal data

**Table:**

C_13_H_10_BrN_3_O_3_	*Z* = 2
*M**_r_* = 336.15	*F*(000) = 336
Triclinic, *P*1	*D*_x_ = 1.762 Mg m^−^^3^
Hall symbol: -P 1	Mo *K*α radiation, λ = 0.71073 Å
*a* = 5.9398 (6) Å	Cell parameters from 26 reflections
*b* = 7.4137 (7) Å	θ = 4.9–13.5°
*c* = 15.897 (2) Å	µ = 3.26 mm^−^^1^
α = 77.846 (9)°	*T* = 295 K
β = 81.613 (7)°	Block, colourless
γ = 68.185 (9)°	0.4 × 0.3 × 0.2 mm
*V* = 633.58 (12) Å^3^	

### Data collection

**Table:**

Siemens P4 diffractometer	1841 reflections with *I* > 2σ(*I*)
Radiation source: fine-focus sealed tube	*R*_int_ = 0.027
Graphite monochromator	θ_max_ = 25.0°, θ_min_ = 2.6°
ω scans	*h* = −6→1
Absorption correction: ψ scan (*XSCANS*; Siemens, 1995)	*k* = −8→8
*T*_min_ = 0.953, *T*_max_ = 0.984	*l* = −18→18
2880 measured reflections	3 standard reflections every 97 reflections
2192 independent reflections	intensity decay: none

### Refinement

**Table:**

Refinement on *F*^2^	Primary atom site location: structure-invariant direct methods
Least-squares matrix: full	Secondary atom site location: difference Fourier map
*R*[*F*^2^ > 2σ(*F*^2^)] = 0.034	Hydrogen site location: inferred from neighbouring sites
*wR*(*F*^2^) = 0.076	H atoms treated by a mixture of independent and constrained refinement
*S* = 1.05	*w* = 1/[σ^2^(*F*_o_^2^) + (0.0258*P*)^2^ + 0.579*P*] where *P* = (*F*_o_^2^ + 2*F*_c_^2^)/3
2192 reflections	(Δ/σ)_max_ < 0.001
186 parameters	Δρ_max_ = 0.30 e Å^−^^3^
0 restraints	Δρ_min_ = −0.41 e Å^−^^3^

### Special details

**Table:**

Geometry. All e.s.d.'s (except the e.s.d. in the dihedral angle between two l.s. planes) are estimated using the full covariance matrix. The cell e.s.d.'s are taken into account individually in the estimation of e.s.d.'s in distances, angles and torsion angles; correlations between e.s.d.'s in cell parameters are only used when they are defined by crystal symmetry. An approximate (isotropic) treatment of cell e.s.d.'s is used for estimating e.s.d.'s involving l.s. planes.
Refinement. Refinement of *F*^2^ against ALL reflections. The weighted *R*-factor *wR* and goodness of fit *S* are based on *F*^2^, conventional *R*-factors *R* are based on *F*, with *F* set to zero for negative *F*^2^. The threshold expression of *F*^2^ > σ(*F*^2^) is used only for calculating *R*-factors(gt) *etc*. and is not relevant to the choice of reflections for refinement. *R*-factors based on *F*^2^ are statistically about twice as large as those based on *F*, and *R*- factors based on ALL data will be even larger.

### Fractional atomic coordinates and isotropic or equivalent isotropic displacement parameters (Å^2^)

**Table:**

	*x*	*y*	*z*	*U*_iso_*/*U*_eq_	
Br	0.90598 (7)	0.62807 (6)	0.37876 (2)	0.04607 (14)	
C1	0.5123 (6)	0.2085 (4)	0.37807 (18)	0.0302 (7)	
C2	0.7390 (6)	0.3054 (5)	0.4494 (2)	0.0401 (8)	
H2A	0.8184	0.2926	0.4978	0.048*	
C3	0.7438 (6)	0.4513 (5)	0.3806 (2)	0.0341 (7)	
C4	0.6228 (6)	0.4664 (5)	0.3101 (2)	0.0373 (8)	
H4A	0.6241	0.5631	0.2622	0.045*	
C5	0.2842 (6)	0.0432 (5)	0.3186 (2)	0.0346 (7)	
C6	0.3460 (6)	0.1063 (4)	0.22515 (19)	0.0307 (7)	
C7	0.5876 (6)	0.0638 (5)	0.1926 (2)	0.0354 (7)	
H7A	0.7120	0.0004	0.2295	0.043*	
C8	0.6427 (6)	0.1155 (5)	0.1058 (2)	0.0345 (7)	
H8A	0.8040	0.0861	0.0840	0.041*	
C9	0.4556 (6)	0.2120 (4)	0.05051 (18)	0.0298 (7)	
C10	0.2152 (6)	0.2496 (5)	0.0825 (2)	0.0360 (8)	
H10A	0.0907	0.3107	0.0455	0.043*	
C11	0.1611 (6)	0.1958 (5)	0.1703 (2)	0.0358 (7)	
H11A	0.0003	0.2202	0.1918	0.043*	
C12	0.5083 (6)	0.2803 (5)	−0.0433 (2)	0.0329 (7)	
C13	0.8156 (7)	0.3160 (5)	−0.1514 (2)	0.0426 (8)	
H13A	0.9781	0.3168	−0.1554	0.064*	
H13B	0.7063	0.4464	−0.1713	0.064*	
H13C	0.8100	0.2271	−0.1864	0.064*	
N1	0.6247 (5)	0.1816 (4)	0.44924 (16)	0.0370 (6)	
N2	0.5040 (5)	0.3454 (4)	0.30896 (16)	0.0384 (7)	
N3	0.3906 (5)	0.0797 (4)	0.38148 (17)	0.0342 (6)	
O1	0.1470 (5)	−0.0495 (4)	0.34026 (15)	0.0528 (7)	
O2	0.7440 (4)	0.2520 (3)	−0.06249 (13)	0.0405 (6)	
O3	0.3578 (5)	0.3532 (4)	−0.09519 (15)	0.0528 (7)	
H3A	0.383 (7)	0.012 (6)	0.431 (3)	0.048 (11)*	

### Atomic displacement parameters (Å^2^)

**Table:**

	*U*^11^	*U*^22^	*U*^33^	*U*^12^	*U*^13^	*U*^23^
Br	0.0571 (2)	0.0489 (2)	0.0434 (2)	−0.03436 (18)	−0.01250 (16)	0.00359 (15)
C1	0.0366 (18)	0.0300 (16)	0.0243 (16)	−0.0142 (14)	−0.0003 (13)	−0.0018 (12)
C2	0.048 (2)	0.052 (2)	0.0272 (17)	−0.0276 (18)	−0.0105 (15)	0.0027 (15)
C3	0.0395 (18)	0.0342 (17)	0.0308 (17)	−0.0177 (15)	−0.0031 (14)	−0.0009 (13)
C4	0.051 (2)	0.0373 (18)	0.0284 (17)	−0.0243 (17)	−0.0072 (15)	0.0032 (14)
C5	0.0408 (19)	0.0347 (17)	0.0309 (17)	−0.0198 (16)	0.0003 (14)	−0.0014 (14)
C6	0.0410 (19)	0.0309 (16)	0.0271 (16)	−0.0214 (14)	−0.0034 (14)	−0.0029 (13)
C7	0.0390 (19)	0.0389 (18)	0.0291 (17)	−0.0163 (15)	−0.0089 (14)	0.0020 (14)
C8	0.0333 (18)	0.0404 (18)	0.0318 (17)	−0.0164 (15)	−0.0023 (14)	−0.0042 (14)
C9	0.0382 (18)	0.0309 (16)	0.0241 (15)	−0.0169 (14)	−0.0059 (13)	−0.0015 (12)
C10	0.0368 (19)	0.0447 (19)	0.0307 (17)	−0.0196 (16)	−0.0090 (14)	−0.0013 (14)
C11	0.0332 (18)	0.0456 (19)	0.0337 (17)	−0.0206 (15)	−0.0035 (14)	−0.0042 (14)
C12	0.0383 (19)	0.0331 (17)	0.0310 (17)	−0.0171 (15)	−0.0070 (15)	−0.0018 (13)
C13	0.050 (2)	0.052 (2)	0.0258 (17)	−0.0240 (18)	−0.0018 (15)	0.0047 (15)
N1	0.0472 (17)	0.0451 (16)	0.0244 (13)	−0.0269 (14)	−0.0052 (12)	0.0033 (12)
N2	0.0566 (18)	0.0393 (15)	0.0264 (14)	−0.0267 (14)	−0.0134 (13)	0.0049 (12)
N3	0.0481 (17)	0.0404 (16)	0.0215 (13)	−0.0281 (14)	−0.0050 (12)	0.0037 (12)
O1	0.0677 (17)	0.0703 (18)	0.0384 (14)	−0.0513 (15)	0.0006 (12)	−0.0005 (12)
O2	0.0412 (14)	0.0524 (14)	0.0252 (11)	−0.0196 (11)	−0.0037 (10)	0.0059 (10)
O3	0.0464 (15)	0.0795 (19)	0.0324 (13)	−0.0284 (14)	−0.0129 (12)	0.0089 (12)

### Geometric parameters (Å, º)

**Table:**

Br—C3	1.887 (3)	C7—H7A	0.9300
C1—N2	1.322 (4)	C8—C9	1.395 (4)
C1—N1	1.339 (4)	C8—H8A	0.9300
C1—N3	1.385 (4)	C9—C10	1.388 (4)
C2—N1	1.329 (4)	C9—C12	1.498 (4)
C2—C3	1.372 (4)	C10—C11	1.392 (4)
C2—H2A	0.9300	C10—H10A	0.9300
C3—C4	1.382 (4)	C11—H11A	0.9300
C4—N2	1.336 (4)	C12—O3	1.200 (4)
C4—H4A	0.9300	C12—O2	1.335 (4)
C5—O1	1.220 (4)	C13—O2	1.448 (4)
C5—N3	1.377 (4)	C13—H13A	0.9600
C5—C6	1.495 (4)	C13—H13B	0.9600
C6—C11	1.379 (4)	C13—H13C	0.9600
C6—C7	1.394 (5)	N3—H3A	0.84 (4)
C7—C8	1.377 (4)		
			
N2—C1—N1	126.4 (3)	C10—C9—C8	120.0 (3)
N2—C1—N3	119.5 (3)	C10—C9—C12	118.8 (3)
N1—C1—N3	114.2 (3)	C8—C9—C12	121.2 (3)
N1—C2—C3	122.2 (3)	C9—C10—C11	119.8 (3)
N1—C2—H2A	118.9	C9—C10—H10A	120.1
C3—C2—H2A	118.9	C11—C10—H10A	120.1
C2—C3—C4	117.3 (3)	C6—C11—C10	120.0 (3)
C2—C3—Br	123.2 (2)	C6—C11—H11A	120.0
C4—C3—Br	119.6 (2)	C10—C11—H11A	120.0
N2—C4—C3	121.5 (3)	O3—C12—O2	123.8 (3)
N2—C4—H4A	119.2	O3—C12—C9	124.5 (3)
C3—C4—H4A	119.2	O2—C12—C9	111.7 (3)
O1—C5—N3	118.9 (3)	O2—C13—H13A	109.5
O1—C5—C6	120.4 (3)	O2—C13—H13B	109.5
N3—C5—C6	120.6 (3)	H13A—C13—H13B	109.5
C11—C6—C7	120.1 (3)	O2—C13—H13C	109.5
C11—C6—C5	119.0 (3)	H13A—C13—H13C	109.5
C7—C6—C5	120.7 (3)	H13B—C13—H13C	109.5
C8—C7—C6	120.2 (3)	C2—N1—C1	116.1 (3)
C8—C7—H7A	119.9	C1—N2—C4	116.5 (3)
C6—C7—H7A	119.9	C5—N3—C1	131.2 (3)
C7—C8—C9	119.8 (3)	C5—N3—H3A	115 (3)
C7—C8—H8A	120.1	C1—N3—H3A	113 (3)
C9—C8—H8A	120.1	C12—O2—C13	116.6 (2)

### Hydrogen-bond geometry (Å, º)

**Table:**

*D*—H···*A*	*D*—H	H···*A*	*D*···*A*	*D*—H···*A*
N3—H3*A*···N1^i^	0.84 (4)	2.14 (1)	2.98 (1)	175 (1)
C13—H13*B*···N2^ii^	0.96	2.58	3.37 (1)	139

Symmetry codes: (i) −*x*+1, −*y*, −*z*+1; (ii) −*x*+1, −*y*+1, −*z*.
